# Biological Treatment Approaches for Degenerative Disc Disease: A Review of Clinical Trials and Future Directions

**DOI:** 10.7759/cureus.892

**Published:** 2016-11-22

**Authors:** Brenton Pennicooke, Yu Moriguchi, Ibrahim Hussain, Lawrence Bonssar, Roger Härtl

**Affiliations:** 1 Department of Neurosurgery, New York-Presbyterian/Weill Cornell Medical Center; 2 Department of Neurosurgery, NewYork-Presbyterian/Weill Cornell Medical Center; 3 Biomedical Engineering, Cornell University

**Keywords:** tissue engineering, degenerative disc disease, degenerative spine, spine, spine surgery, gene therapy, biomolecular therapy

## Abstract

Biologic-based treatment strategies for musculoskeletal diseases have gained traction over the past 20 years as alternatives to invasive, costly, and complicated surgical interventions. Spinal degenerative disc disease (DDD) is among the anatomic areas being investigated among this group, notably due to its high incidence and functional debilitation. In this review, we report the literature encompassing the use of biologic-based therapies for DDD. Articles published between January 1995 and November 2015 were reviewed, with a subset meeting the primary and secondary inclusion criteria of clinical trial results that could be sub-classified into bimolecular, cell-based, or gene therapies, as well as studies investigating the utility of allogeneic and tissue-engineered intervertebral discs. Ongoing clinical trials that have not yet published results are also mentioned to present the current state of the field. This exciting area has demonstrated positive and encouraging results across multiple strategies; thus, future bimolecular and regenerative techniques and understanding will likely lead to an increase in the number of human clinical trials assessing these therapies.

## Introduction and background

The radiographic findings of degenerative disc disease (DDD) can be found in 40% of individuals younger than 30 and in more than 90% of individuals older than 50 years of age [1-2]. While the majority of these imaging findings are part of the normal aging process, a subset of patients will present with symptomatic nerve root compression and chronic back pain ultimately requiring surgical intervention [3-4]. DDD can be treated pharmacologically with opiates, steroids, or non-steroidal anti-inflammatory drugs. Likewise, other conservative measures such as physical therapy and corticosteroid injections are frequently prescribed. However, these measures do not treat the underlying cause of the degenerative process and do not slow the natural progression of the disease. In progressively symptomatic patients not responsive to conservative measures, surgery is indicated. The type of intervention is based on the underlying pathology and symptomatology, ranging from discectomy to placement of an interbody graft for bony fusion. While controversial, reports of reherniation, pseudarthrosis, and adjacent segment disease can lead to recurrent symptoms and reoperations [5-6]. Prosthetic total disc replacement (TDR) devices are now being used in clinical practice as an alternative to fusion; however, multiple studies have shown that TDR devices also alter spine biomechanics significantly enough to lead to adjacent segment degeneration (ASD) [6-7].

Given the potential complications of these surgical interventions, attention to biologic-based therapies for DDD has gained traction. Trials of gene therapy, in addition to cellular- and acellular-based transplantations have been described in degenerative knee and metacarpophalangeal arthritis, with promising results [8-11]. Thus, translation to their spinal counterparts has been an intense area of research. However, the inherent multi-factorial nature of DDD presents a challenge for optimal treatment strategies. Biomechanical, immunologic, environmental, and genetic factors influence DDD, and their complex interactions are not well understood. Biomolecular therapies (e.g. genes and proteins), cell-based therapies, (e.g. stem cells and chondrocytes), and total disc replacement (allogeneic or tissue-engineered) are the broad categories of research in biologics for DDD. Depending on the stage of degeneration, different treatment strategies have been employed with varying degrees of success. In the present review, we present the published and unpublished clinical studies of biological disc repair and discuss future directions in this regenerative field.

## Review

### Materials and methods

The PubMed, Google Scholar, Embase, ClinicalTrials.gov, and Cochrane Library databases were searched for relevant studies from January 1995 to November 2015. The following keywords were queried in combination with *intervertebral disc* or *degenerative disc disease:* *gene therapy,* *cell therapy, molecular therapy, stem cell, mesenchymal stem cell, disc cell, nucleus pulposus cell, disc chondrocyte, disc regeneration, and tissue engineering*. After the initial search, 213 studies were identified. These studies’ results were reviewed, duplicates were identified, and only relevant studies were included. The primary inclusion criterion was the presence of clinical results on disc regeneration. The secondary inclusion criterion was the ability to categorize into one of the following categories: biomolecular therapy, cell-based therapy, gene therapy, and tissue engineered intervertebral disc (IVD). These categories were generated based on the literature that is available on *in vivo *animal studies on biologic therapy for DDD. For each study, we identified the type of study, the questionnaire-based subjective assessment on pain, and radiologic outcome measures. Following this advanced filtering, a total of 24 studies were included for discussion.

### Pathophysiology of disc degeneration

**Figure 1:**
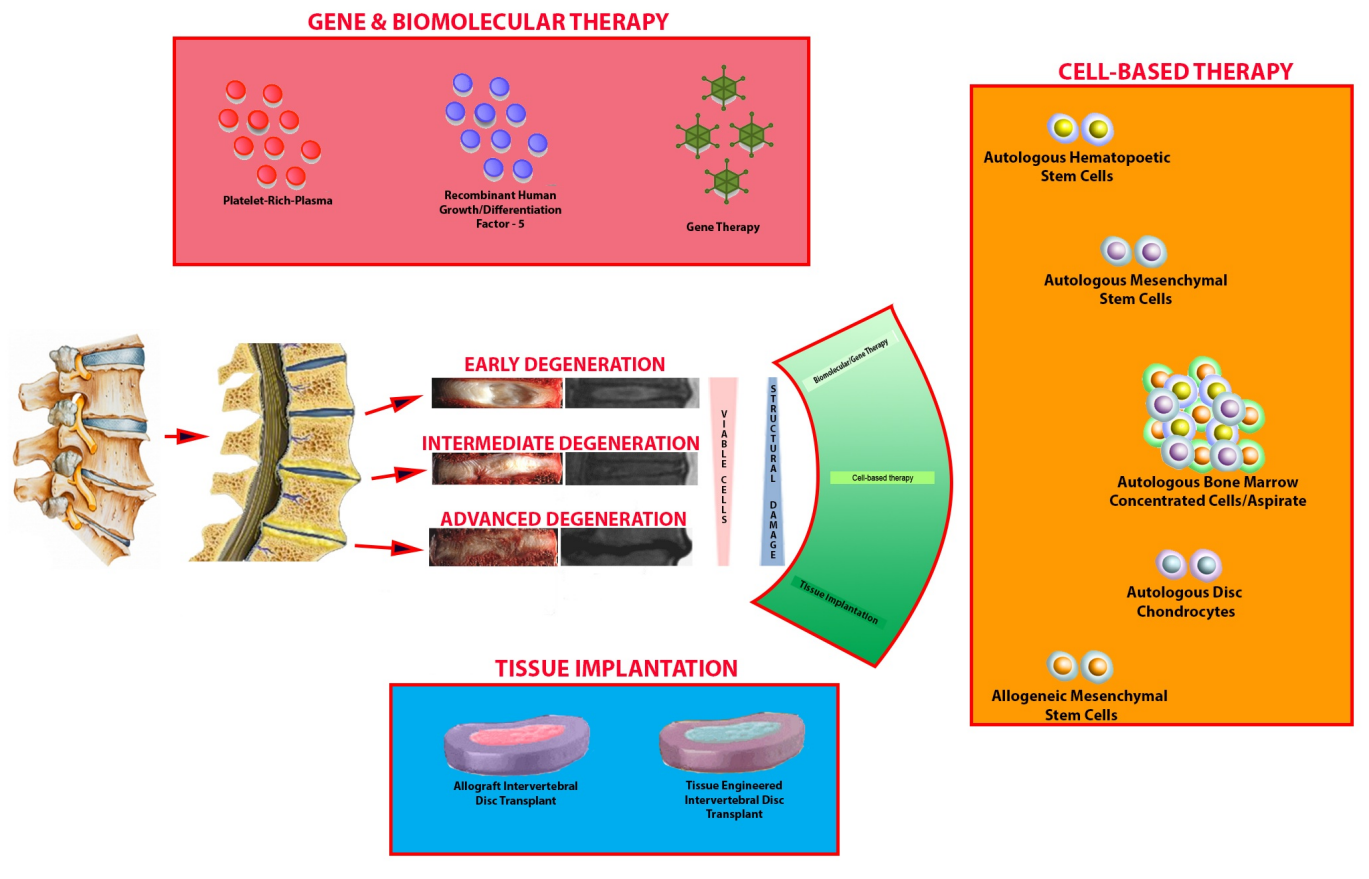
Schematic for Degenerative Disc Disease and Biologic Therapies

The IVD is composed of the nucleus pulposus (NP) surrounded by the annulus fibrosis (AF), sandwiched between cartilaginous endplates at the junction to the vertebral bodies located above and below the IVD. The NP is mainly composed of proteoglycans and type II collagen, which allows for the retention of water increasing the IVD’s ability to handle axial loading. The surrounding AF is more stiff and is primarily composed of type I collagen. With increasing age, the water content within the IVD decreases and results in the NP being less resilient to mechanical stressors. This progressive decrease in resiliency leads to NP fissures, which can extend into the AF. This marks the initial stages of degenerative destruction of the IVD, endplates, and associated vertebral bodies (Figure 1).

### Biomolecular therapy for disc regeneration

As previously stated, DDD is a multifactorial process including the progressive decline in NP hydration due to loss of proteoglycans and collagen. This decreased hydration results in loss of mechanical tension in the AF collagen fibers and results in abnormal spinal axial loading forces and segmental instability. These minor changes in stress forces on the spine can result in the development of neck or back pain and narrowing of the spinal canal over time. In early stage degeneration, the disc undergoes an imbalance of anabolic and catabolic factors that leads to extracellular matrix (ECM) degradation [12]. Specifically, the diseased state of decreased anabolism and increased catabolism can be modified by recombinant proteins and genes to regenerate expression of target molecules. The goal would be to facilitate ECM synthesis and promote NP rehydration and nutrition. The following section will review recent studies on biomolecules used to treat disc degeneration (Table 1).

**Table 1:** Clinical Trials Using Biologic-based Therapies for Degenerative Disc Disease

Primary Researcher	Biologic Therapy	Study Design	Number of Patients	Follow-Up (M)	Findings	Journal
Meisel, et al.	Autologous Disc Chondrocyte Transplantation (EuroDisc)	Multicenter prospective, randomized, controlled, non-blinded study	28	24	Patients who received ADCT had lower pain scores as tabulated by the OPDQ than control. Patients who received ADCT had retention of better hydration of the disc than control, but no change in disc height	EuroSpine J 2006, 2008
Orozco, et al.	Autologous Bone Marrow Mesenchymal Cell	Pilot Study/ Case Series	10		Improvement in pain, disability, and disc hydration	Transplantation 2001
Yoshikawa, et al.	Autologous Bone Marrow Mesenchymal Cell	Pilot Study/Case Series	2	24	Both patients showed improvement in pain? And intensity of T2-weighted MRIs	Spine 2010
Haufe SMW, et al.	Hematopoietic Stem Cell	Pilot Study/Case Series	10	12	No improvement in back pain	Stem Cells Dev. 2006
Coric D, et al.	Allogeneic Juvenile Chondrocytes (NuQu)	Pilot Study/ Case Series	15	12	ODI, NRS SF-36 improvement from baseline with 89% of patients showing some improvement on MRI	JNS 2013
Berlemann, et al.	Injectable Biomimetic Nucleus Hydrogel	Pilot Study/Case Series	14	24	Significant improvement in leg and back pain after micro-discectomy	Euro Spine 2009
Ruan, et al.	Total Disc Replacement with Allogeneic IVD	Pilot Study/Case Series	5	60	The allograft engrafted the disc space without apparent immunoreaction; 4 out 5 implanted disc spaces preserved their range of motions after disc implantation	Lancet 2007
Pettine, et al.	Injection of Autologous Bone Marrow Concentrate Cells	Pilot Study/Case Series	26	12	Improvement in pain scores prominently in patients with higher CFU-F concentrations. Rehydration of the discs observed (n=8)	Stem Cells 2015

Recombinant Human Growth/Differentiation Factor-5

Growth/differentiation Factor-5 (GDF-5) is a member of the transforming growth factor-b (TGF-b) superfamily and the bone morphogenetic protein (BMP) subfamily and is known to influence the growth and differentiation of various tissues, including the intervertebral disc. In vitro and in vivo experiments have shown that human recombinant GDF-5 (rhGDF-5) can stimulate gene expression and synthesis of ECM proteins such as type II collagen and aggrecan [13]. In September 2014, a multicenter, randomized, double-blind, placebo-controlled clinical trial was conducted to evaluate the safety and tolerability of intradiscal rhGDF-5 in subjects with early lumbar disc degeneration [14]. Twenty-four subjects with persistent low back pain with at least three months of non-surgical therapy at one suspected symptomatic lumbar level (L3/4 to L5/S1) were included in the study. The subjects received a discogram to confirm that they had at least one symptomatic level attributable to DDD. Additionally, they all had an Oswestry Disability Index (ODI) for low back pain of 30 or greater and Visual Analog Scale (VAS) of four or greater. Subjects who had an abnormal neurological exam at baseline, radicular pain due to anatomic nerve root compression, extravasation of contrast during the discogram, or suspected symptomatic facet joints and/or severe facet joint degeneration were all excluded from the trial. The subjects were evaluated through a 12-month period followed by annual telephone contact at 24 and 36 months for subject health status follow-up. The secondary outcome was the preliminary effectiveness of intradiscal rhGDF-5 as compared to placebo at the same time frame. The results of this study have not been published yet.

Platelet-rich Plasma

Platelet-rich-plasma (PRP) is a fraction of plasma that can be produced by centrifugal separation of whole blood. A platelet contains the vast majority of biologically active molecules required for blood coagulation, such as adhesive proteins, coagulation factors, and protease inhibitors [15]. In addition to these factors, PRP also carries a number of factors that are known to increase collagen content, accelerate epithelial regeneration, promote angiogenesis, improve wound healing, and stimulate IVD metabolism [16-18]. More specifically, PRP includes growth factors such as TGF-b, platelet-derived growth factor (PDGF), epidermal growth factor (EGF), vascular endothelial growth factor, and insulin-like growth factor-1 (IGF-1). These factors have been shown to enhance cell viability, stimulate ECM metabolism, and stimulate proliferation of IVD cells [19]. The presence of these factors within PRP lead to the proposal to use it as an intradiscal therapy to stimulate regeneration or at least slow the progression of degeneration within a diseased IVD. A 2012 published abstract described the safety and feasibility of intradiscal PRP injection in reducing low back pain in patients with DDD [20]. This abstract included 12 individuals with one or more lumbar discs (L3/4 to L5/S1) with >3 months of low back pain without leg pain, degenerative changes on magnetic resonance imaging (MRI), and at least one symptomatic disc confirmed using a standardized provocative discography procedure. The participants were followed for six months with interval radiographic and clinical assessment. The PRP releasate solution, which was isolated from clotted PRP, was injected (2.0 ml) into the center of the nucleus pulposus under fluoroscopic guidance. At one month, pain scores (as assessed by VAS and Japanese Orthopaedic Association (JOA)) showed a significant decrease and was sustained for 12 months after treatment. However, disc height indices were not significantly changed over the follow-up period (p=0.12) and the mean T2 hydration assessments did not change significantly after treatment.

Further evidence for PRP therapy in DDD was augmented in 2015 by a prospective, randomized controlled study assessing intradiscal PRP injections in discogenic-mediated low back pain. This study demonstrated improvements in pain and function in patients as early as eight weeks post-treatment and was sustained for up to one year [21]. Forty-seven adults with chronic (≥ 6 months) moderate to severe lumbar discogenic pain, unresponsive to conservative treatment were randomized to receive an intradiscal PRP injection or an intradiscal contrast agent after a provocative discography. Twenty-nine subjects were randomized to the treatment group and eighteen subjects were randomized to the control group. Data on pain, physical function, and overall satisfaction were collected at one week, four weeks, eight weeks, six months, and one year. The study had a 92% follow-up rate at eight-week time points or longer and found statistically significant improvement in participants who received intradiscal PRP injections with regard to pain, function, and overall satisfaction. Although this was a novel study with very promising results, there were a number of limitations in this study. One limitation of the study was the limited follow-up time of only eight weeks for the control group, thus limiting the assessment of how long the effects of the PRP injections are seen. In other words, if the PRP injected groups and control groups had no statistical difference in their pain and functionality scores at one year after the treatment, it could be stated that the PRP injection provided some short-term symptomatic relief but did not provide any longer-term symptomatic relief. Additionally, the participants in the study were not standardized by the degree of disc degeneration. Therefore, some participants had more disc degeneration and larger protrusions than others, which likely increased the variability of the responses. Lastly, there was no radiographic assessment of the degenerated disc. Thus this study did not show that injecting PRP affected the natural progression of the disc degeneration. This would have been critical information for the use of biomolecular therapy for DDD.

Cell-based Therapy for Disc Regeneration

Biomolecular therapy likely has limited efficacy in discs with higher grades of degeneration, as the number of cells responsive to injected genes and proteins decline with progression degeneration. Cell-based therapy is the optimal treatment strategy in mid-stage degeneration because it directly addresses the decreased number of viable chondrocytes and stem cells within the diseased disc space (Table 2).

**Table 2:** Unpublished Clinical Trials Using Biologic-based Therapies for Degenerative Disc Disease

PI/ Sponsor	Title of Trial	Biologic Therapy	Study Design	Number of Patients	Follow-up (M)	Status
ISTO Technologies, Inc.	A Study Comparing the Safety and Effectiveness of Cartilage Cell Injected Into the Lumbar Disc as Compared to a Placebo	Allogeneic juvenile chondrocytes (NuQu) in fibrin carrier.	Double-blind, Randomized, Phase 2	44	24	Phase II done
Mesoblast, Ltd.	Safety and Preliminary Efficacy Study of Mesenchymal Precursor Cells (MPCs, Mesoblast) in Subjects With Lumbar Back Pain	6 or 18 million MPCs (Mesoblast) in a hyaluronic acid carrier	Double-blind, Randomized, Phase 2	100	36	Phase II done
Red de Terapia Celular	Treatment of Degenerative Disc Disease With Allogeneic Mesenchymal Stem Cells (MSV) (Disc_allo)	25 millions MSC in 2 ml of saline	Double-blind, Randomized, Phase 1, 2	24	12	Ongoing
K-Stemcell Co., Ltd.	Autologous Adipose Tissue Derived Mesenchymal Stem Cells Transplantation in Patient With Lumbar Intervertebral Disc Degeneration	Autologous Adipose Tissue derived MSCs	Non-randomized, Open label	8	6	Ongoing
Bioheart, Inc.	Adipose Cells for Degenerative Disc Disease	Adipose tissue-derived stem cells suspended in platelet-rich plasma	Non-randomized, Open label	100	12	Ongoing
DePuy Spine	Intradiscal rhGDF-5 (BMP14) for Early Stage Lumbar DDD	rhGDF-5	Double-blind, Randomized, Phase 1, 2	38	36	Ongoing
Mochida J, et al.	Intradiscal rhGDF-5 (BMP14) for Early Stage Lumbar DDD	Autologous NP cells from fusion, co-cultured with bone marrow MSCs	Case Series	10	24	Ongoing
Lutz, et al. HSS	Lumbar Intradiscal PRP injections	Single injection of PRP	Double-blind, Randomized Controlled study	72	6	Complete
Akeda, et al.	Intradiscal Injection of PRP-releasate for the Treatment of Lumbar Disc Degeneration	Injection of the soluble releasate isolated from clotted PRP	Case-Series	6	6	Complete

Autologous Disc Chondrocytes

The use of autologous disc cells is an alternative approach to repair damaged or chronically inflamed tissue by addressing multiple propagators of degeneration at once. From 2002 to 2006, the first study of autologous disc chondrocyte transplantation (ADCT) in a large group enrolled patients in a multicenter prospective, randomized, controlled, non-blinded study to compare the safety and efficacy of ADCT. Known as the EuroDISC trial, this study compared ADCT plus discectomy to discectomy alone to evaluate if ADCT mitigated postoperative pain. In this study, 28 participants between the ages of 18 and 60 with a body mass index below 28 and 1-level lumbar canal stenosis requiring surgical intervention were included. Patients with multiple levels of stenosis requiring operative intervention, discs with sclerosis, edema, or modic changes of grade II or III, and/or focal spondylolisthesis were excluded. Twelve patients received percutaneous ADCT 12 weeks following discectomy and 16 patients received only discectomy. The patients were followed for two years and assessed using the Oswestry low back back pain disability questionnaire (OPDQ) as the primary criterion. Secondary criteria included MRI and X-ray evaluation. They found that the cell therapy group had continual improvement in their OPDQ after the initial surgery compared to no improvement in the control group, and this results persisted at the two-year follow-up [22]. Additionally, the analysis of the fluid content of the IVD as measured by T2 signal intensity on MRI showed 16% more retention of fluid within the nucleus pulposus. However, comparison of the mean IVD heights revealed no difference between the groups. These results were very promising; however, the final results and analysis have not been published yet. This ADCT system is currently manufactured and marketed in Germany but has not been approved by the FDA and thus is not available in the United States.

Autologous Mesenchymal Stem Cells

Mesenchymal stem cells (MSCs) derived from bone marrow are one of the most well-studied cell types in regenerative medicine due to their accessibility and expandability in ex vivo conditions. Additionally, the anti-inflammatory effects of MSCs have been demonstrated in numerous animal models of injury including myocardial infarction, renal ischemia, reperfusion injury, burn wounds, and osteoarthritis [23-24]. There have been multiple attempts to introduce mesenchymal precursor cells or MSC into the intradisc space to treat chronic low back pain. In 2010, Yoshikawa, et al. analyzed the regenerative ability of autologous MSCs in markedly degenerated IVDs of two patients with chronic low back pain, radiculopathy, and paresthesias [25]. MSCs isolated from bone marrow aspirate were coupled with collagen sponges and grafted percutaneously to the degenerated IVD following partial laminotomy. Two years after surgery, both patients had significant symptomatic relief as assessed by VAS, and T2-weighted MRIs showed high signal within the treated IVDs indicating high NP hydration without progressive degeneration. Although the study was limited by a very small sample size, it did serve as a proof of principle showing that autologous MSCs may play a role in the treatment of DDD.

In a similar regard, Orozco, et al. published a study of 10 patients with chronic back pain diagnosed due to DDD with autologous MSCs injected directly into the NP [26]. These patients were followed for one year and pre- and post-treatment MRIs were obtained. Both lumbar pain and disability were strongly reduced at three months after MSC transplantation, followed by modest additional improvement at six and 12 months. The short form-36 (SF-36) life quality questionnaire revealed by the end of treatment a significant improvement of the physical component with no change of the mental component. They also found marginal improvement in disc hydration of the treated levels at one year but no significant change in disc height.

Despite these results, the major challenge to MSCs therapy is the pain and cost associated with harvesting these cells. Since MSCs are found in adipose tissue, this poses an attractive source for harvesting and subsequent transplant due to the low-risk accessibility, and current trials evaluating their efficacy in intradiscal applications are ongoing [27-28].

Autologous Hematopoietic Stem Cells

Hematopoietic stem cells are thought to be useful to treat disc degeneration due to their differentiation and proliferative capacities. However, as applies to most cells, the oxygen-poor environment poses a challenge for biologic therapies in spinal disc disease. In 2006, Haufe, et al. described a study utilizing hyperbaric oxygenation in 10 patients following percutaneous intradiscal injection of autologous hematopoietic precursor stem cells (HSCs) [29]. Ultimately, none of the 10 patients achieved any improvement of their discogenic pain after one year and eight out of the 10 patients underwent surgical treatment within one year study completion. Although disappointing, the study provided insight into the use of non-mesenchymal lineage cells in DDD.

Autologous Bone Marrow Aspirate/Bone Marrow Concentrated Cells

**Figure 2:**
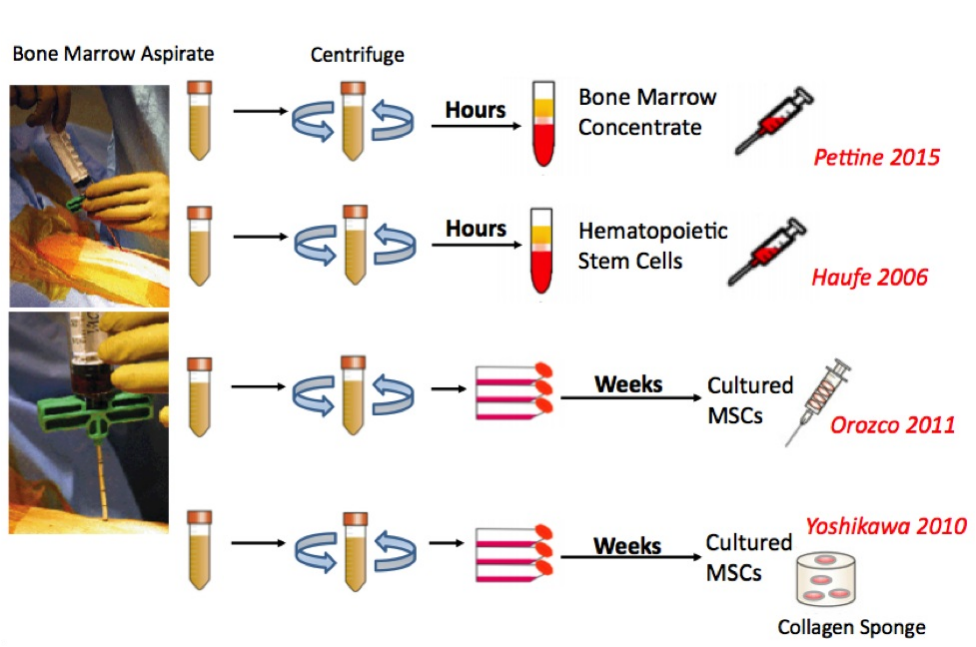
Preparation of Bone Marrow-Based Cell Therapy

Harvesting, culturing, and expanding autologous chondrocytes or MSCs is an expensive process and requires the patient to undergo at least two procedures since the harvested cells must be cultured and expanded in a lab for weeks prior to transplantation (Figure 2).

Bone marrow concentrated cells (BMCs) contain multiple stem and progenitor cells, including mesenchymal stem cells (MSCs) and can be autotransplanted at the time of surgery, precluding a second delayed intervention. Pettine, et al. evaluated the use of autologous, nonexpanded BMCs to treat moderate to severe discogenic low back pain [30]. The study included 26 participants who had 55 mL of bone marrow aspirate collected from the iliac crest and subsequently injected in the intradisc space of a diseased level. Significant improvement in ODI, VAS, and modified Pfirrmann score were reported at three, six, and 12 months posttreatment. Upon closer examination of the bone marrow aspirate, the authors discerned that patients who received >2,000 colony-forming unit-fibroblasts (CFU-F) per milliliter of bone marrow aspirate had statistically significant improvement in pain scores compared to those patients with <2000 CFU-F/ml. The exact mechanism by which the increased concentration of CFU-F lead to improvement in discogenic back pain remains unclear and is currently the focus of follow-up studies.

Allogeneic Mesenchymal Precursor Cells/Mesenchymal Stem Cells

**Figure 3:**
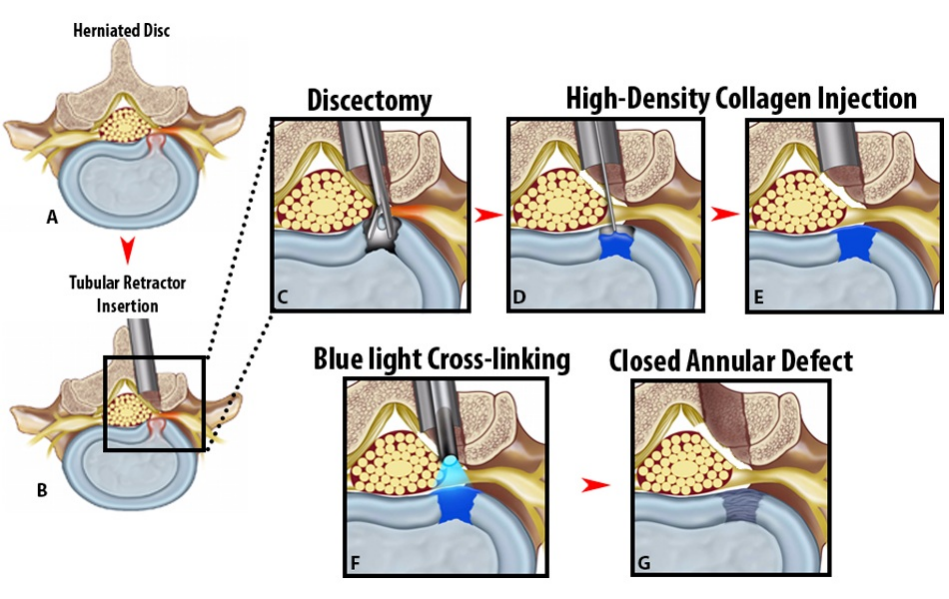
Injectable High-Density Collagen Gel

Allogeneic stem cells are another method of biologic treatment for DDD being investigated that is attractive due to the low costs of harvesting and preclusion of secondary procedures. Currently, two clinical trials are underway evaluating their effects. Mesoblast, a private cellular medicine company, is conducting a double-blinded, placebo-controlled Phase 2 clinical trial of 100 patients with DDD randomized to received intradisc injection of saline, hyaluronic acid, or allogeneic mesenchymal precursor cells (MPCs) of varying concentrations in a hyaluronic acid [31]. Similarly, a private group in Spain is conducting Phase 2 clinical trials for allogeneic MSCs in 12 patients compared with controls [32]. Clinical and radiographic endpoints will be collected and while results have not been published yet, the utility of allogeneic stem cells remain an intense area of investigation. Finally, biologic strategies for annular repair incorporating annulus fibrocytes and MSCs following surgical intervention may aid in slowing the progressive, degenerative nature of disc disease after manipulation (Figure 3). These strategies are still being developed in pre-clinical animal models, but promising preliminary data will likely see them transition to human clinical trials in the coming years.

Allogeneic Juvenile Disc Chondrocytes

Similar to the previous reports of autologous disc chondrocyte transplantation, groups have assessed the efficacy of allogeneic juvenile chondrocytes into degenerated discs. In a prospective cohort study, Coric, et al. demonstrated that NuQu, an injectable percutaneous fibrin-based delivery of juvenile chondrocytes, helps improve low back pain refractory to conservative measures [33]. Fifteen patients, with a mean age of 40 years, were treated with a single percutaneous delivery of 1-2 mL of NuQu containing 10^7 ^juvenile chondrocyte cells/ml within a fibrin carrier. Ten of the 13 patients exhibited significant clinical and radiographic improvements, and interestingly eight of nine patients with the posterior annular tears had resolution of significant improvement in the degree of posterior annular tear, suggesting ongoing remodeling of the injured AF.

Gene Therapy for DDD

Injection of biologic molecules into the disc allows for direct delivery of protein products to address the causes of degenerative disc disease at the source. However, these biological molecules and many cell-based therapies have short half-lives which preclude their longevity following injection. Gene therapy has emerged as a potential solution to this, which comprises of delivery of recombinant genes into cells. The recombinant gene can then incorporate with the DNA of the host cell allowing for long-term expression of either a proanabolic or anticatabolic protein to facilitate healing of the degenerated disc space [34]. This strategy requires identification of relevant genes that play a role in the disc degeneration cascade and then the ability to deliver those potentially therapeutic cells to the disc space. Though the evidence generated from *in vivo* animal models has been promising, no clinical trials have been conducted using this technique in humans. Since viral carriers are often used for transfection, serious concerns involving unexpected mutations create challenges for Food and Drug Administration (FDA) approval and patient inclusion. Nonetheless, gene therapy has shown promising results in other neurologic and orthopedic diseases and thus remains an area of interest for biologic treatment strategies in DDD. 

Allogeneic and Tissue Engineered IVD Transplantation for DDD

At present, both *in vivo* and *in vitro* experiments using tissue engineered IVD are in their preliminary states. One promising study published by Ruan, et al. included five patients with cervical spondylosis who underwent transplantation of fresh-frozen composite disc allografts following discectomy [35]. The disc allografts were harvested from 13 previously healthy organ donors aged 20–30 years. Within two hours of cardiac arrest, the cervical spine was removed en-bloc from C3 to T1 under sterile conditions. The patients were then implanted with the harvested allografts and were followed with serial flexion-extension X-rays and MRIs. Of note, these patients did not receive any immunosuppressive agents and were simply monitored with weekly measurements of erythrocyte sedimentation rate, C-reactive protein, and peripheral blood counts to assess for organ rejection. At the five-year follow-up, the motion and stability of the implanted spinal segment was preserved, but only two of the five implanted grafts showed signs of adequate NP hydration on T2-weighted MRI. All five patients reported improvement in symptoms at the five-year follow-up and none encountered immunoreaction. This proof-of-concept study has created a new alternative to biologic treatment of DDD; however, challenges are expected. First the supply of organ donors will need to be established and criteria for which donors are most suitable still need to be clearly defined. Furthermore, while none of these patients encountered immunologic reactions, the vast majority of transplantation recipients require some form of immunosuppression, which increases the risk of opportunistic infections and malignancies in a subset of patients.

There have not been any human clinical trials for the implantation of tissue engineered IVD (TE-IVDs). However, there has been one human pilot study for using a biomimetic protein polymer that mimics the NP. Berlemann, et al. used NuCore® injectable nucleus (Spine Wave, Inc., CT, USA), which is a protein polymer hydrogel that mimics the properties of the natural nucleus to treat post-discectomy patients [36]. The polymer chain is composed of silk and elastin components designed for both elasticity and toughness. This hydrogel is injected as a fluid through the annular defect as a replacement for nuclear tissue lost to herniation and microdiscectomy. Fourteen patients with single-level herniated discs that were unresponsive to conservative therapy had NuCore® hydrogel injected following microdiscectomy that was allowed to cure/harden over five minutes. Ultimately, the group found significant improvement in leg and back pain scores, and functional scores (as assessed by ODI) following the procedure. Postoperative MRI showed stable position of the implants, and radiographic measurements showed restoration of disc height. This study serves as the first of its kind to use an engineered polymer to replace a native biologic structure following surgical removal.

## Conclusions

The information elucidated from various studies on biologics presents an exciting new area of research for DDD. Adaptations and modifications of similar modalities used in degenerative joint arthritis may one day be applicable to spinal disc disease as an upfront therapy. Future research into the use of viral vector gene therapy, RNA interference, and micro RNAs may provide fruitful alternatives to cell-based and whole IVD-based therapies, but significant challenges need to be addressed prior to translation to human clinical trials.

## References

[1] Andersson GB (1999). Epidemiological features of chronic low-back pain. Lancet.

[2] Cheung KM, Karppinen J, Chan D, Ho DW, Song Y-Q, Sham P, Cheah KS, Leong JC, Luk KD (2009). Prevalence and pattern of lumbar magnetic resonance imaging changes in a population study of one thousand forty-three individuals. Spine.

[3] Brinjikji W, Luetmer PH, Comstock B, Bresnahan BW, Chen LE, Deyo RA, Halabi S, Turner JA, Avins AL, James K, Wald JT, Kallmes DF, Jarvik JG (2015). Systematic literature review of imaging features of spinal degeneration in asymptomatic populations. AJNR Am J Neuroradiol.

[4] Borenstein DG, O'Mara JW, Jr. Jr., Boden SD, Lauerman WC, Jacobson A, Platenberg C, Schellinger D, Wiesel SW (2001). The value of magnetic resonance imaging of the lumbar spine to predict low-back pain in asymptomatic subjects : a seven-year follow-up study. J Bone Joint Surg Am.

[5] Sugawara T, Itoh Y, Hirano Y, Higashiyama N, Mizoi K (2009). Long term outcome and adjacent disc degeneration after anterior cervical discectomy and fusion with titanium cylindrical cages. Acta Neurochir (Wien).

[6] Maldonado CV, Paz RD, Martin CB (2011). Adjacent-level degeneration after cervical disc arthroplasty versus fusion. Eur Spine J.

[7] Kelly MP, Mok JM, Frisch RF, Tay BK (2011). Adjacent segment motion after anterior cervical discectomy and fusion versus Prodisc-c cervical total disk arthroplasty: analysis from a randomized, controlled trial. Spine.

[8] Mease PJ, Hobbs K, Chalmers A, El-Gabalawy H, Bookman A, Keystone E, Furst DE, Anklesaria P, Heald AE (2009). Local delivery of a recombinant adenoassociated vector containing a tumour necrosis factor α antagonist gene in inflammatory arthritis: a phase 1 dose-escalation safety and tolerability study. Ann Rheum Dis.

[9] Evans CH, Robbins PD, Ghivizzani SC, Wasko MC, Tomaino MM, Kang R, Muzzonigro TA, Vogt M, Elder EM, Whiteside TL (2005). Gene transfer to human joints: progress toward a gene therapy of arthritis. Proc Natl Acad Sci USA.

[10] Marcacci M, Berruto M, Brocchetta D, Delcogliano A, Ghinelli D, Gobbi A, Kon E, Pederzini L, Rosa D, Sacchetti GL, Stefani G, Zanasi S (2005). Articular cartilage engineering with Hyalograft. Clin Orthop Relat Res.

[11] Kon E, Delcogliano M, Filardo G, Pressato D, Busacca M, Grigolo B, Desando G, Marcacci M (2010). A novel nano-composite multi-layered biomaterial for treatment of osteochondral lesions: technique note and an early stability pilot clinical trial. Injury.

[12] Sivan SS, Hayes AJ, Wachtel E, Caterson B, Merkher Y, Maroudas A, Brown S, Roberts S (2014). Biochemical composition and turnover of the extracellular matrix of the normal and degenerate intervertebral disc. Eur Spine J.

[13] Chujo T, An HS, Akeda K, Miyamoto K, Muehleman C, Attawia M, Andersson G, Masuda K (2006). Effects of growth differentiation factor-5 on the intervertebral disc− in vitro bovine study and in vivo rabbit disc degeneration model study. Spine.

[14] NIH NIH (2016). A multicenter, randomized, double-blind, placebo controlled, clinical trial to evaluate the safety, tolerability and preliminary effectiveness of 2 doses of intradiscal rhGDF-5 (single administration) for the treatment of early stage lumbar disc degeneration. Clinicaltrials.gov.

[15] Harrison P, Cramer EM (1993). Platelet α-granules. Blood Rev.

[16] Ross R (1987). Platelet-derived growth factor. Annual review of medicine.

[17] Declair V (1999). The importance of growth factors in wound healing. Ostomy Wound Manage.

[18] Carter CA, Jolly DG, Worden CE, Hendren DG, Kane CJ (2003). Platelet-rich plasma gel promotes differentiation and regeneration during equine wound healing. Exp Mol Pathol.

[19] Gruber HE, Norton HJ, Hanley Jr EN (2000). Anti-apoptotic effects of IGF-1 and PDGF on human intervertebral disc cells in vitro. Spine (Phila Pa 1976).

[20] Akeda K IT, Ohishi K, Masuda K, Uchida A, Sakakibara T, Kasai Y, Sudo Sudo (2016). Intradiscal injection of autologous platelet-rich plasma for the treatment of lumbar disc degeneration -preliminary prospective clinical trial for discogenic low back pain patients. ORS.

[21] Tuakli-Wosornu YA, Terry A, Boachie-Adjei K, Harrison JR, Gribbin CK, LaSalle EE, Nguyen JT, Solomon JL, Lutz GE (2016). Lumbar intradiskal platelet-rich plasma (PRP) injections: a prospective, double-blind, randomized controlled study. PM R.

[22] Meisel HJ, Siodla V, Ganey T, Minkus Y, Hutton WC, Alasevic OJ (2007). Clinical experience in cell-based therapeutics: disc chondrocyte transplantation: a treatment for degenerated or damaged intervertebral disc. Biomol Eng.

[23] Black LL, Gaynor J, Adams C, Dhupa S, Sams AE, Taylor R, Harman S, Gingerich DA, Harman R (2008). Effect of intraarticular injection of autologous adipose-derived mesenchymal stem and regenerative cells on clinical signs of chronic osteoarthritis of the elbow joint in dogs. Vet Ther.

[24] Black LL, Gaynor J, Gahring D, Adams C, Aron D, Harman S, Gingerich DA, Harman R (2007). Effect of adipose-derived mesenchymal stem and regenerative cells on lameness in dogs with chronic osteoarthritis of the coxofemoral joints: a randomized, double-blinded, multicenter controlled trial. Vet Ther.

[25] Yoshikawa T, Ueda Y, Miyazaki K, Koizumi M, Takakura Y (2010). Disc regeneration therapy using marrow mesenchymal cell transplantation: a report of two case studies. Spine.

[26] Orozco L, Soler R, Morera C, Alberca M, Sánchez A, García-Sancho J (2011). Intervertebral disc repair by autologous mesenchymal bone marrow cells: a pilot study. Transplantation.

[27] NIH NIH (2016). NIH: autologous adipose tissue derived mesenchymal stem cells transplantation in patient with lumbar intervertebral disc degeneration. ClinicalTrialsgov.

[28] NIH NIH (2016). NIH: an open Label, non-randomized, multi-center study to assess the safety and effects of 
autologous adipose-derived stromal cells delivered intra-discally in patients with degenerative disc disease. ClinicalTrialsgov.

[29] Haufe SM, Mork AR (2006). Intradiscal injection of hematopoietic stem cells in an attempt to rejuvenate the intervertebral discs. Stem.

[30] Pettine KA, Murphy MB, Suzuki RK, Sand TT (2015). Percutaneous injection of autologous bone marrow concentrate cells significantly reduces lumbar discogenic pain through 12 months. Stem Cells.

[31] NIH NIH (2016). Safety and preliminary efficacy study of mesenchymal precursor cells (MPCs) in subjects with lumbar back pain. clinicaltrialsgov.

[32] UNIo UNIo (2016). Treatment of degenerative disc disease with allogenic mesenchymal stem cells (MSV) (Disc_allo). NCT01860417.

[33] Coric D, Pettine K, Sumich A, Boltes MO (2012). Prospective study of disc repair with allogeneic chondrocytes: presented at the 2012 Joint Spine Section Meeting. J Neurosurg Spine.

[34] Kadow T, Sowa G, Vo N, Kang JD (2014). Molecular basis of intervertebral disc degeneration and herniations: what are the important translational questions?. Clin Orthop Relat Res.

[35] Ruan D, He Q, Ding Y, Hou L, Li J, Luk KD (2007). Intervertebral disc transplantation in the treatment of degenerative spine disease: a preliminary study. The Lancet.

[36] Berlemann U, Schwarzenbach O (2009). An injectable nucleus replacement as an adjunct to microdiscectomy: 2 year follow-up in a pilot clinical study. Eur Spine J.

